# Supplementary material for: RadiomiX for Radiomics Analysis: Automated Approaches to Overcome Challenges in Replicability
Source: Diagnostics (Basel). 2025 Aug 5;15(15):1968. doi: 10.3390/diagnostics15151968 (PMC12345906; doi:10.3390/diagnostics15151968)
Supplement: Supplementary file 1 [file diagnostics-15-01968-s001.zip › diagnostics-3747478-supplementary.pdf]

# RadiomiX for Radiomics Analysis: Automated Approaches to Overcome Challenges in Replicability

Journal Name: Diagnostics (MDPI)
Author list: Harel Kotler, Luca Bergamin, MSc, Fabio Aiolli, PhD, Angela Grassi, MD, Giulia Pasello, MD, PHD, Alessandra Ferro, MD, Elena Scagliori, MD, Francesca Caumo, MD, Gisella Gennaro, PHD
Corresponding author: Gisella Gennaro, gisella.gennaro@iov.veneto.it
Radiology and Radiotherapy, Veneto Institute of Oncology IOV – IRCCS, 35128, Padua, Italy

# Supplemental material S1 - Pipeline parameters and hyperparameters

## Feature selection methods used

Feature selection is pivotal in radiomics, as it addresses the curse of dimensionality, reduces potential overfitting, and improves model interpretability. The following methods were evaluated for their suitability in radiomics, where small sample sizes and high feature counts necessitate robust and efficient approaches.

### Spearman’s Rank Correlation

Spearman’s rank correlation is a non-parametric method that assesses the relationship between radiomic features and the target variable by ranking their values and calculating the correlation between these ranks.

The rank correlation coefficient (ρ) is calculated as

$$\rho = 1 - (6 * \Sigma d^2)/(n * (n^2 - 1))$$

where (d_i) is the difference between the ranks of corresponding values, and (n) is the number of observations.

This method is commonly used in radiomics as it is more resilient to outliers, does not assume a specific data distribution, and captures non-linear relationships.
On the other hand, this method cannot detect feature redundancy, potentially selecting correlated features unnecessarily. This limitation can be addressed by following this method with another feature selection method adapted for handling redundancy and feature interactions.

To automate the correlation process, our specific implementation included Spearman's Correlation followed by Kruskal Wallis test and Dunn's non parametric post hoc-test. Spearman's correlation coefficient was calculated to identify the correlation between the features. Correlation clusters were built using Numpy and NetworkX, with a correlation cutoff set to 0.8. Each cluster was then tested using Kruskal-Wallis test and Dunn's nonparametric post hoc test.

Spearman, C. (January 1904). "The Proof and Measurement of Association between Two Things" . The American Journal of Psychology. 15 (1): 72–101. doi:10.2307/1412159.

## Minimum Redundancy, Maximum Relevance (mRMR)

mRMR is a multivariate filter method that selects features by maximizing their relevance to the target variable while minimizing redundancy among selected features. Relevance is measured using mutual information ($I(f_i;c)$), between a feature ($f_i$) and the target (c), while redundancy is measured as the mutual information, $I(f_i;f_j)$, between pairs of features. The algorithm iteratively selects features that optimize this trade-off.

The mRMR criterion can be described as:

$$max\ S(\frac{1}{|S|}\sum_{fi\epsilon S} \quad I(fi;c) - \frac{1}{|S|}\sum_{fi,fj\epsilon S} \quad I(fi;fj))$$

Where (S) is the selected feature set, $I(f_i;c)$ is the mutual information between feature ($f_i$) and the target (c), and $I(f_i;f_j)$ is the mutual information between features.

mRMR is widely used in radiomics for its ability to reduce redundancy among highly correlated features. However, its computational intensity may limit its use in datasets with thousands of features unless combined with initial filtering methods.

Peng, H., Long, F., & Ding, C. (2005). Feature selection based on mutual information: Criteria of max-dependency, max-relevance, and min-redundancy. IEEE Transactions on Pattern Analysis and Machine Intelligence, 27(8), 1226–1238. DOI: 10.1109/TPAMI.2005.159

## Least Absolute Shrinkage and Selection Operator (LASSO)

LASSO is an embedded feature selection method that integrates feature selection into the training of a linear regression model by adding an L1 regularization term to the loss function. This penalty shrinks the coefficients of less important features to zero.

The LASSO optimization problem is:

$$min\,\beta\{(\frac{1}{2N}\sum_{i=1}^{N}\quad(y_{\mathrm{i}}\,-\beta_0-x_i^T\beta)^2+\lambda\sum_{j=1}^{p}\quad|\beta_{\mathrm{j}}|\}$$

Where $\beta$ are the regression coefficients, $\lambda$ is the regularization parameter, and $\beta_{\mathrm{j}}$ is the L1 norm.

LASSO is one of the most commonly used feature selection methods in radiomics due to its ability to handle high-dimensional data and select sparse models. However, it relies on a linearity assumption between features and the target as well as sensitivity to the L1 parameter tuning.

Tibshirani, R. (1996). Regression shrinkage and selection via the lasso. Journal of the Royal Statistical Society: Series B (Methodological), 58(1), 267–288. Link

## Random Forest (RF) for Feature Selection

Random Forest is an ensemble feature selection method that combines multiple decision trees to assess feature importance through intrinsic variable ranking. The method calculates feature importance by measuring the average decrease in node impurity (typically Gini impurity or entropy) when a feature is used for splitting across all trees in the forest.

The RF feature importance is calculated as:

$$Importance(X_{\mathrm{j}})\;=\;(1/B)\sum_{\beta=1}{}^{\mathrm{B}}\sum_{\mathrm{i}}\in T_{\beta}\,I(v_{\mathrm{i}}\;=\;X_{\mathrm{j}})\;\times\;p_{\mathrm{i}}\;\times\;\Delta I_{\mathrm{i}}$$

Where B is the number of trees, $T_\beta$ represents nodes in tree b, $I(v_i = X_j)$ indicates if feature $X_j$ is used at node i, $p_i$ is the proportion of samples reaching node i, and $\Delta I_i$ is the impurity decrease at node i.

RF can handle non-linear relationships and tends to be more resistant to outliers. However, it can be biased toward features with more categories and may struggle with highly correlated features, which are common in radiomic datasets.

Breiman, L. (2001). Random forests. Machine learning, 45(1), 5-32.

## Feature selection methods implementation

| Feature selection method | Implementation | Hyperparameter grid |
|---|---|---|
| Correlation | pandas.DataFrame.corr(method='spearman')<br>scikit_posthocs.posthoc_dunn<br>nx.from_edgelist()<br>nx.algorithms.find_cliques<br>scipy.stats.kruskal | Correlation cutoff = 0.8 |
| Maximum Relevance — Minimum Redundancy (mRMR) | mrmr.mrmr_classif using the specific version in https://github.com/smazzanti/mrmr and a predefined total of features per specific combination (ranging from 1 to 100).<br>In the LLN dataset , as only 82 features were available, all features were selected as the highest option. | K: 1, 2, 5, 10, 15, 25 |
| Least Absolute Shrinkage and Selection Operator (LASSO) | sklearn.linear_model.Lasso | Alpha: 0.001, 0.0001, 0.00001 |

| | | |
|---|---|---|
| Random Forest (RF) | sklearn.ensemble.RandomForestClassifier | Top_K: 1, 2, 5, 10, 15, 25 |

# Classifier used

## Logistic Regression (LR)

Logistic Regression is a linear classifier that models the probability of class membership using the logistic function, which maps a linear combination of features to probabilities between 0 and 1. The model minimizes the log-likelihood loss function using optimization techniques such as gradient descent.

The LR optimization problem is:

$$min\, \beta\{-\sum_{i=1}^{N}[y_i log(p(x_i)) + (1-y_i) log(1-p(x_i))]\}$$

Where $p(x_i) = 1/(1 + e\^(-\beta_0 - x_i^T\beta))$ and β are the regression coefficients.

LR is frequently used in radiomics due to its simplicity, which translates into its interpretability. However, it assumes linear relationships between features and log-odds, and is sensitive to outliers and multicollinearity, which are common in radiomic datasets.

Cox, D. R. (1958). The regression analysis of binary sequences. Journal of the Royal Statistical Society: Series B (Methodological), 20(2), 215–232.

## K-Nearest Neighbors (KNN)

KNN is a non-parametric, instance-based classifier that assigns class labels based on the majority vote of the k nearest neighbors in the feature space using distance metrics like Euclidean distance.

The KNN decision rule is:

$$\hat{y} = argmax\_c \sum_{i} \in N_k(x)\, I(y_i = c)$$

Where $N_k(x)$ represents the k nearest neighbors of instance x, and I is the indicator function.

KNN is less commonly used in radiomics due to computational intensity and sensitivity to high-dimensional data and redundant features. It is prone to overfitting with small sample sizes typical in radiomic studies, requiring careful feature curation and k selection.

Fix, E., & Hodges, J. L. (1951). Discriminatory analysis: Nonparametric discrimination: Consistency properties. USAF School of Aviation Medicine.

## Naive Bayes (NB)

Naive Bayes is a probabilistic classifier that applies Bayes' theorem with the assumption of conditional independence between features given the class. It calculates posterior probabilities for each class and selects the class with the highest probability.

The NB classification rule is:

$$\hat{y} = argmax\_c \, P(c)\prod_{j=1}^{p} P(x_j|c)$$

Where P(c) is the prior probability and $P(x_j|c)$ is the likelihood of feature j given class c.

This classifier often struggles in radiomics analysis due to correlated features, which are common in radiomics, that challenges the independence assumption.

Domingos, Pedro; Pazzani, Michael (1997). "On the optimality of the simple Bayesian classifier under zero-one loss". Machine Learning. 29 (2/3): 103–137. doi:10.1023/A:1007413511361.

## Random Forest (RF)

In the previous section RF was described for feature selection. While having the same core function, its implementation as a classifier leads to different advantages and disadvantages. Therefore, though repeating the previously explained method, we explain RF again in its entirety in the classification context.

Random Forest is an ensemble classifier that combines multiple decision trees trained on bootstrapped data subsets with random feature selection at each split. The final prediction is determined by majority voting across all trees, reducing variance and improving generalization.

The RF prediction is:

$$\hat{y} = mode\{h_t(x)\}_{t=1}^{T}$$

Where $h_t(x)$ is the prediction of the t-th tree and T is the total number of trees.

RF is popular in radiomics due to its ability to handle high-dimensional data and capture non-linear relationships. However, it can be computationally intensive and less interpretable than simpler models. Furthermore, despite addressing overfitting through ensemble averaging, overfitting is still quite possible due to the small sample size typical for radiomics.

Breiman, L. (2001). Random forests. Machine learning, 45(1), 5-32.

## Extreme Gradient Boosting (XGBoost)

XGBoost is an optimized gradient boosting classifier that sequentially builds decision trees, where each tree corrects errors from previous iterations. It incorporates L1 and L2 regularization terms and uses second-order derivatives for more precise optimization.

The XGBoost objective function is:

$$L^{(t)} = \sum_{i=1}^{n} l(y_i, \hat{y}_i^{(t-1)} + f_t(x_i)) + \Omega(f_t)$$

Where l is the loss function, $f_t$ is the t-th tree, and $\Omega(f_t)$ is the regularization term.

XGBoost achieves high performance on structured data but may be overly complex for small radiomic datasets, increasing overfitting risk. While its regularization capabilities help, it requires extensive hyperparameter tuning to reach its full potential.

Chen, T., & Guestrin, C. (2016). XGBoost: A scalable tree boosting system. Proceedings of the 22nd ACM SIGKDD International Conference on Knowledge Discovery and Data Mining, 785–794.

## Adaptive Boosting (AdaBoost)

AdaBoost is an ensemble classifier that combines multiple weak learners by iteratively training them on weighted versions of the data, where misclassified instances receive higher weights in subsequent iterations. The final prediction is a weighted combination of all weak learner outputs.

The AdaBoost weight update rule is:

$$w_i^{(m+1)} = w_i^{(m)} exp(-\alpha_m y_i h_m(x_i))$$

Where $w_i$ is the instance weight, $\alpha_m$ is the weak learner weight, and $h_m(x_i)$ is the m-th learner prediction.

AdaBoost is a robust classifier, yet can be sensitive to noise and outliers. Similarly to XGBoost, it requires careful tuning and validation to achieve its full potential.

Freund, Y., & Schapire, R. E. (1997). A decision-theoretic generalization of on-line learning and an application to boosting. Journal of Computer and System Sciences, 55(1), 119–139.

## Classifiers implementation

| Classifier | Implementation | Hyperparameter grid |
|---|---|---|
| Logistic Regression (LR) | sklearn.linear_model.LogisticRegression | C: 0.05, 0.1, 0.5, 1, 2, 4, 10<br>penalty: l1, l2, None<br>solver: saga, sag, lbfgs, liblinear<br>classifier__max_iter: 10, 50, 100, 500, 1000, 5000 |
| Support Vector Machine (SVM) | sklearn.svm.SVC | probability=True<br>kernel: linear, rbf, poly<br>C: 0.01, 0.05, 0.1, 0.5, 1<br>gamma: 1, 0.5, 0.1, 0.05, 0.01, scale |
| K-Nearest Neighbors (KNN) | sklearn.neighbors.KNeighborsClassifier | n_neighbors: 1, 3, 5, 7, 9<br>weights: uniform, distance<br>p: 1, 2<br>algorithm: auto, ball_tree, kd_tree, brute |
| Naive Bayes classifier (NB) | sklearn.naive_bayes.MultinomialNB | alpha: 0.1, 0.5, 1.0, 2.0, 4.0<br>fit_prior: True |
| Random Forest (RF) | sklearn.ensemble.RandomForestClassifier | n_estimators: 50, 100, 200, 250, 300<br>max_features: sqrt, log2 |

| | | |
|---|---|---|
| | | max_depth: 10, 15, None<br>criterion: gini, entropy<br>Bootstrap: True, False,<br>min_samples_leaf: 1,2,4 |
| Adaptive Boosting classifier (AdaBoost) | sklearn.ensemble.AdaBoostClassifier | n_estimators: 50, 100, 200, 250, 300<br>learning_rate: 0.3, 1.0, 1.5<br>algorithm: SAMME, SAMME.R' |
| Extreme Gradient Boost Classifier (XGBoost) | xgboost.XGBClassifier | enable_categorical=True<br>tree_method="hist"<br>learning_rate: 0.05, 0.1, 0.3, 0.5<br>max_depth: 0, 6, 10<br>n_estimators: 50, 100, 200, 250, 300<br>subsample: 0.6, 0.8,1 |

## Scalers

Scaler options for each dataset:

| Scaler | Implementation | Hyperparameter Grid |
|---|---|---|
| MinMaxScaler | sklearn.preprocessing.MinMaxSclaer | - |
| StandardScaler | sklearn.preprocessing.StandardScaler | - |

| | | |
|---|---|---|
| RobustScaler | sklearn.preprocessing.Robust Scaler | - |
| None | - | - |

True/False hyperparameter was added to each scaler for the option of using power transformation based on sklearn.preprocessing.PowerTransformer using "box-cox" or 'yeo-johnson' based on the amount of negative columns.

## Class balancing

Both RandomOverSampler and SMOTE were implemented as oversampling options based on imblearn.over_sampling. A set of oversampler options were tested in each pipeline based on the original number of samples in each class given in the sampling_strategy parameter.

| Dataset | Original class 1 size | Resampled class 1 size | Original class 2 size | Resampled class 2 size |
|---|---|---|---|---|
| LLN | 524 | 525, 530 | 314 | 315, 368, 420, 475, 505, 510, 525, 530 |
| SLN | 377 | 380, 400 | 359 | 360, 365, 370, 375, 378, 380, 388, 390, 395, 400 |
| MBC | 127 | 128, 130, 140 | 101 | 110, 128, 130, 140 |
| CHE | 86 | 90, 100 | 38 | 40, 50, 80, 90, 100 |

# Supplemental material S2 - Input and use description, optimal pipeline settings, and hyperparameters

## Input description

RadiomiX takes csv files as input with samples as rows and radiomic features as columns. It also requires the target column and the sample ID column name.

No graphical interface is required; all options are passed as standard CLI arguments or specified in a single JSON parameter file (for custom settings). Input images are not supplied directly to RadiomiX—only the feature matrix exported from any radiomics extraction tool is needed, keeping imaging-format dependencies (e.g., DICOM) outside the scope of the modelling pipeline.

## Execution

RadiomiX is executed from the command line the following way:

```
python radiomix.py
    --features  path/to/feature_table.csv
    --target    Target
    --idcol     ID
    --params    fast_params
```

There are 2 pre-defined param schemes, one with a very small amount of parameters (fast_params ≈ 15 min run-time) and one with a significantly larger and extensive parameters list (full_params ≈ 6 h on a 10-core CPU). There is also the possibility to add a custom params json file as input.

# Optimal settings and hyperparameters

| Dataset | Classifier | Feature selection | Mean AUC | 95% CI | Mean ACC | 95% CI | Mean F1 | over sampler | scaler | Classifier hyperparams |
|---|---|---|---|---|---|---|---|---|---|---|
| LLN | RF | RF | 0.850 | 0.734, 0.919 | 0.785 | 0.694, 0.863 | 0.828 | Random 530:530 | None | n_estimators: 300, Bootstrap: False, Criterion: Entropy, Max Depth: None, Max_features: Log2 |
| SLN | LR | MRMR,KW,LASSO | 0.845 | 0.772, 0.915 | 0.754 | 0.653, 0.830 | 0.741 | Random 380:360 | MinMax | C: 1, max_iter: 1000, penalty: l2, solver: saga |
| MBC | RF | LASSO | 0.889 | 0.7678, 0.979 | 0.833 | 0.714, 0.952 | 0.850 | Random 130:130 | None | n_estimators: 50, Bootstrap: False, Criterion: Entropy, Max Depth: None, Max_features: Sqrt |
| CHE | RF | LASSO | 0.837 | 0.649,0.967 | 0.730 | 0.583, 0.909 | 0.717 | SMOTE 80,80 | None | n_estimators: 50, Bootstrap: True, Criterion: Entropy, Max Depth: 15, Max_features: Sqrt |

# Selected radiomic features

| Dataset | Selected radiomic features |
|---|---|
| LLN | 'GLCM_Correl_25HUgl', 'GLRLM_GLN_25HUgl', 'NGTDM_Busyne_25HUgl', 'GLCM_InfCo1_25HUgl', 'GLCM_InfCo2_25HUgl', 'GLRLM_RLN_25HUgl', 'GLSZM_ZoneHiGl_25HUgl', 'GLSZM_SzVarianc_25HUgl' 'N.voxels', 'GLCM_IDN_25HUgl', 'NGTDM_Streng_25HUgl', 'GLSZM_GlNonUnif_25HUgl', 'GLSZM_SzoneHiGl_25HUgl', 'GLCM_AutoCorrel_25HUgl', 'GLCM_IDMN_25HUgl', 'GLSZM_SzNonUnif_25HUgl', 'GLRLM_SRHGLE_25HUgl', 'NGTDM_Contra_25HUgl', 'GLCM_sumAvg_25HUgl', 'GLRLM_HGLRE_25HUgl', 'GLSZM_SmallZone_25HUgl', 'FOS_Kurt', 'GLSZM_GlVarianc_25HUgl', 'GLRLM_RP_25HUgl', 'NGTDM_Complex_25HUgl' |
| SLN | 'Annulus_GLSZM_SzoneLoGl_LHL_25HUgl', 'Annulus_GLCM_Angsmo_LLL_25HUgl', 'Annulus_NGTDM_Coarse_HHH_25HUgl', 'Annulus_GLSZM_ZoneLoGl_LLL_25HUgl', 'Lesion_GLRLM_LRLGLE_25HUgl', 'Annulus_FOS_Ener_LLL_25HUgl', 'Annulus_NGTDM_Coarse_HHL_25HUgl', 'Lesion_GLCM_IDN_HLH_25HUgl', 'Lesion_NGTDM_Coarse_HLL_25HUgl', 'Annulus_GLCM_InfCo1_HLH_25HUgl', 'Lesion_GLCM_IDMN_HLL_25HUgl', 'Lesion_GLCM_InfCo2_LHL_25HUgl', 'Annulus_GLCM_MxProb_25HUgl', 'Lesion_GLCM_InfCo1_HLH_25HUgl', 'Annulus_FD_var_25HUgl', 'Lesion_GLCM_invVar_HLL_25HUgl', 'Annulus_GLRLM_LRLGLE_25HUgl', 'Lesion_GLCM_Correl_25HUgl', 'Lesion_GLCM_IDMN_HHL_25HUgl', 'Annulus_GLCM_InfCo2_HLH_25HUgl', 'Annulus_GLCM_difEnt_LLL_25HUgl', 'Annulus_GLCM_InfCo1_HLL_25HUgl', 'Annulus_FD_sd_LLL_25HUgl', 'Lesion_GLCM_IDN_LHH_25HUgl', 'Lesion_NGTDM_Contra_LHH_25HUgl' |
| MBC | 'Kurtosis_PET', 'Skewness_CT', 'SUV_max' |
| CHE | 'GLRLM_LGRE', 'GLZLM_SZLGE', 'GLRLM_SRLGE', 'NGLDM_Busyness', 'GLZLM_ZP', 'NGLDM_Contrast', 'HISTO_Skewness', 'NGLDM_Coarseness' |
